# Negative Feedback Regulation of HIV-1 by Gene Editing Strategy

**DOI:** 10.1038/srep31527

**Published:** 2016-08-16

**Authors:** Rafal Kaminski, Yilan Chen, Julian Salkind, Ramona Bella, Won-bin Young, Pasquale Ferrante, Jonathan Karn, Thomas Malcolm, Wenhui Hu, Kamel Khalili

**Affiliations:** 1 Department of Neuroscience Center for Neurovirology, Lewis Katz School of Medicine at Temple University, 3500 N. Broad Street, 7th Floor Philadelphia, PA 19140, USA; 2 Department of Radiology University of Pittsburgh School of Medicine Pittsburgh, PA 15219, USA; 3 Microbiology and Clinical Microbiology, Department of Biomedical, Surgical and Dental Sciences, University of Milan, Milan, Italy; 4 Department of Molecular Biology and Microbiology, Case Western Reserve University, Cleveland, OH 44106, USA; 5 Excision Biotherapeutics, Inc., 3624 Market Street, #514, Philadelphia, PA 19104, USA.

## Abstract

The CRISPR/Cas9 gene editing method is comprised of the guide RNA (gRNA) to target a specific DNA sequence for cleavage and the Cas9 endonuclease for introducing breaks in the double-stranded DNA identified by the gRNA. Co-expression of both a multiplex of HIV-1-specific gRNAs and Cas9 in cells results in the modification and/or excision of the segment of viral DNA, leading to replication-defective virus. In this study, we have personalized the activity of CRISPR/Cas9 by placing the gene encoding Cas9 under the control of a minimal promoter of HIV-1 that is activated by the HIV-1 Tat protein. We demonstrate that functional activation of CRISPR/Cas9 by Tat during the course of viral infection excises the designated segment of the integrated viral DNA and consequently suppresses viral expression. This strategy was also used in a latently infected CD4+ T-cell model after treatment with a variety of HIV-1 stimulating agents including PMA and TSA. Controlled expression of Cas9 by Tat offers a new strategy for safe implementation of the Cas9 technology for ablation of HIV-1 at a very early stage of HIV-1 replication during the course of the acute phase of infection and the reactivation of silent proviral DNA in latently infected cells.

Soon after HIV-1 infection, the viral genome becomes integrated into the host chromosome and is rapidly expressed in CD4+ T-cells. HIV-1 replication leads to drastic depletion of CD4+ T-cells^1^,^2^. Often, after the acute phase of infection, the virus enters a new phase called latency, where the integrated proviral DNA continues to be expressed and viral replication proceeds at very low levels. Under these circumstances, the weakened immune system caused by persistent viral replication progresses towards AIDS and the development of a broad range of opportunistic infections that eventually lead to death within three years if untreated^3^. At the molecular level, expression of the viral genome and its replication both at the acute and chronic stages is controlled by the viral promoter that spans 450 nucleotides of the 5′-long terminal region (LTR)^4^,^5^. Cooperativity occurs between a series of cellular transcriptional factors that recognize DNA sequences within the U3 region of the 5′-LTR and the HIV-1 immediate early transcription activator, Tat, that interacts with the TAR RNA sequence positioned within the leader region of the viral transcript. These interactions are required for the robust initiation and efficient elongation of transcription from integrated copies of the viral DNA^6^,^7^. While current anti-retroviral drugs have been effective in suppressing viral infection cycles, they have yet to contain any components that inhibit viral gene expression at the transcriptional level, supporting the notion that the integrated copies of the virus may continue to express the viral genome, albeit at very low levels, in HIV-1+ patients under ART^8^,^9^. Indeed, expression of viral genes drastically elevates upon cessation of ART and allows production of viral early regulatory proteins such as Tat to orchestrate productive replication of the viral genome.

In recent years, more attention has been paid to the development of effective and safe strategies toward a cure for HIV-1/AIDS. In this respect, several approaches, including elimination of latently infected cells that serve as viral reservoirs by activation of the dormant virus and boosting immune cells, known as the shock and kill strategy. While this strategy was initially promising, it has shown limited efficacy and inconsistent outcomes^10^,^11^,^12^. More recently, the discovery of novel gene editing technologies prompted several laboratories to explore possibilities for inactivating viral DNA by introducing mutations within the various regions of the viral genome and/or cellular genes that support HIV-1 infection^13^,^14^,^15^. Our laboratory has employed the CRISPR/Cas9 technology and developed an HIV-1 specific gene editing molecule that, for the first time, excised the entire HIV-1 genome between the 5′- and 3′-LTRs from the host chromosomes of latently infected cells and protected the cells from re-infection^13^,^16^,^17^. The method of excision included use of multiplex specific gRNAs that recognize various regions of the 5′- and 3′-LTR DNA sequences and the Cas9 endonuclease, which introduces breaks on double-stranded DNA at the sites that are complementary to the gRNAs^16^,^17^. After removal of viral DNA, the residual cellular DNA re-joins by cellular DNA repair^13^,^14^,^16^,^17^. CRISPR/Cas9 gene editing using a multiplex of different gRNAs that introduce InDel mutations as well as excision of segments of the viral DNA has also been utilized by several other laboratories^18^,^19^. The use of a multiplex of gRNAs for editing the HIV-1 genome by CRISPR technology is particularly critical in order to alleviate any concern related to the emergence of resistant virus against the initial gRNA treatment. In addition to CRISPR/Cas9 technology, more recently, recombinase based procedures have been developed with the ability to edit the HIV-1 DNA sequence from the host genome^20^.

In the studies presented here, we have refined our gene editing technique and have developed a new strategy that allows conditional activation of the CRISPR/Cas9 at an early stage of viral reactivation by the HIV-1 transcriptional activator, Tat. This new strategy permanently ablates virus replication prior to productive viral replication by removing a segment of the viral gene spanning the viral promoter and/or the viral coding sequence. Further, this strategy will alleviate any concerns due to unforeseen complications that may arise by unnecessary and persistent expression of Cas9 at high levels in cells.

## Results

**Figure 1:**
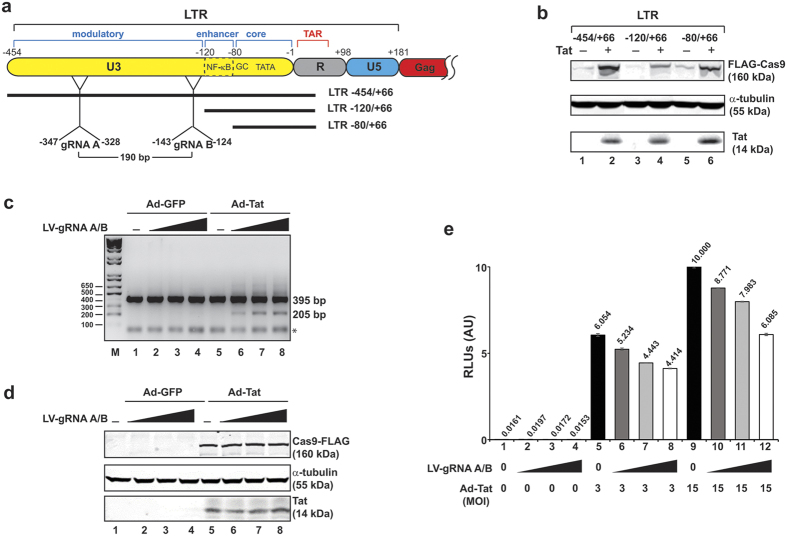
Expression of Cas9 by the HIV-1 LTR promoter is stimulated by Tat leading to cleavage of the viral promoter in the presence of gRNA. (**a**) Schematic presentation of the full-length HIV-1 LTR and the various regulatory motifs within the enhancer and core regions, and the partial Gag gene. The extent of LTR deletion mutants that are created for expression of Cas9 is depicted. The positions of the gRNA target sequence and their distance from each other is shown. (**b**) Co-transfection of TZM-bl cells with pX260-LTR-Cas9 containing the full-length LTR (−454/+66) or its various mutants (−120/+66 or −80/+66) along with a plasmid expressing Tat (pCMV-Tat) increased the level of Cas9 production as tested by Western blot (top panel). Expression of housekeeping α-tubulin (middle panel) and Tat (bottom panel) are shown. (**c**) Infection of TZM-bl cells with adenovirus expressing GFP or Tat followed by transduction with lentivirus expressing Cas9 by the LTR_−80/+66_ promoter and gRNAs A/B by the U6 promoter at three different MOI of 2, 4 and 8 led to cleavage of the integrated HIV-1 LTR promoter DNA sequence and the appearance of a 205 bp DNA fragment in the TZM-bl cells (as tested by PCR and DNA gel analysis). (**d**) SDS-PAGE illustrating the level of Cas9, α-tubulin and Tat protein expressed in TZM-bl cells as described in Panel C. E. Luciferase assay illustrating transcriptional activity of the integrated HIV-1 LTR in TZM-bl cells after various treatments as described in Panel c.

We placed the coding DNA sequence corresponding to the Cas9 gene in a pX260 expression vector, containing three different segments of the HIV-1 promoter spanning the U3 and R regions of the 5′-LTR to identify the minimal DNA elements of the viral promoter that remain responsive to Tat, yet lacks the sequences corresponding to gRNAs A and B that are initially used for editing HIV-1 DNA (Fig. 1a). After verification of our cloning strategy by DNA sequencing of each construct, expression of Cas9 by each vector and the level of their response to Tat was examined in TZM-bl cells co-transfected with pX260-LTR-Cas9 and CMV-Tat. Results from Western blot revealed activation of Cas9 expression by all three constructs including the plasmid encompassing the minimal DNA promoter sequence positioned between −80 to +66 (Fig. 1b). A basal level of the minimal promoter (−80/+66) led to low levels of expression of Cas9 in these cells. This was particularly important for our studies as the promoter sequence resides outside of the DNA sequences corresponding to gRNAs A and B (Fig. 1b). Next, a DNA fragment corresponding to LTR_(−80/+66)_-Cas9 was cloned into a lentiviral vector (LV) and used to transduce TZM-bl cells to assess the effect of Tat protein on the editing of integrated copies of HIV-1 DNA expressing the luciferase reporter gene. Results from PCR amplification of the LTR revealed the detection of 205 bp DNA fragment in cells expressing gRNAs A and B and Tat protein (Fig. 1c, compare lanes 1–5 to lanes 6–8). The position of the primers used for PCR amplification and the expected amplicons are illustrated in Supplemental Fig. 1. Expression of Cas9, Tat and α-tubulin (control for equal loading) are shown in Fig. 1d.

**Figure 2:**
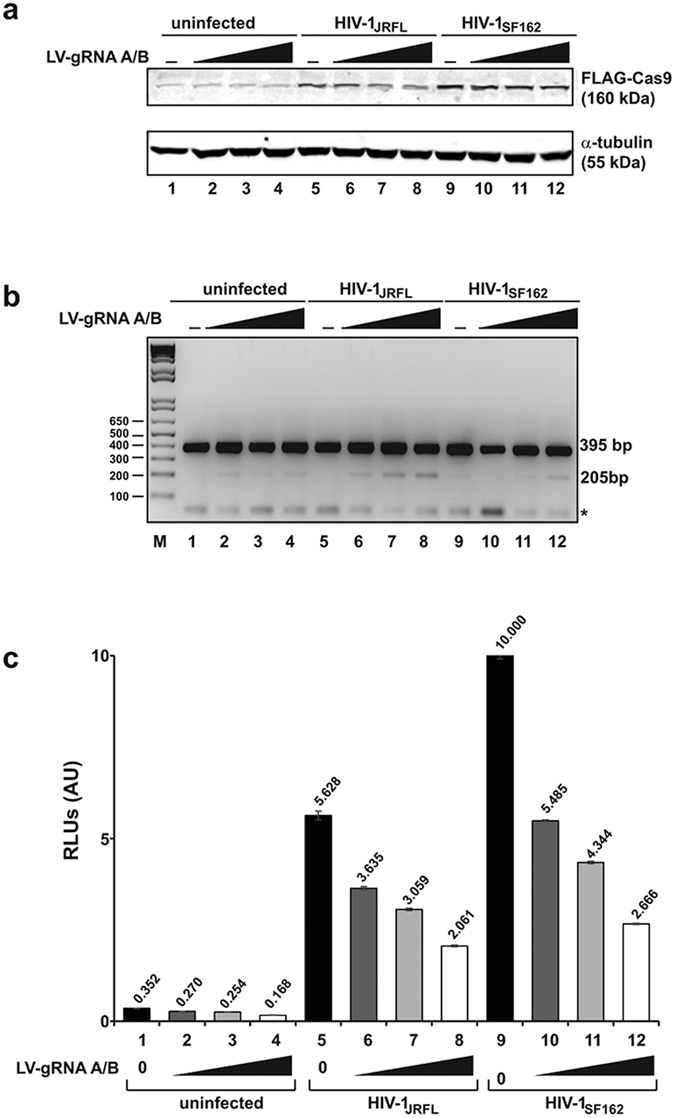
HIV-1 infection stimulates cleavage of integrated viral DNA upon induction of Cas9. The LTR_−80/+66_-Cas9 reporter TZM-bl cell line transduced with three different MOI (2, 4, and 8) of lentivirus expressing gRNA A/B (LV-gRNA A/B) or control (empty LV) was infected with HIV-1_JRFL_ or HIV-1_SF162_, and after 48 hours, cells were harvested and protein expression was determined by Western blot (**a**), the level of integrated HIV-1 LTR cleavage upon induction of Cas9 after viral infection was detected by PCR/DNA gene analysis (**b**) and transcriptional activity of the integrated HIV-1 promoter was evaluated by luciferase reporter assay (**c**).

Next, we examined the impact of the viral DNA excision on viral promoter activity by luciferase assay. Results show a gradual decrease in luciferase activity upon activation of Cas9 by Tat, corroborating the results from DNA assay, indicating that the cleavage of DNA causes inhibition of viral promoter activity in these cells (Fig. 1e). In follow-up studies, we investigated the activation of Cas9 upon infection of TZM-bl cells by HIV-1. To this end, the LTR_(−80/+66)_-Cas9 reporter TZM-bl cells were transduced by LV-gRNAs A/B for 24 hours, after which cells were infected with HIV-1_JRFL_ or HIV-1_SF162_ at three different MOIs. After 48 hours, cells were harvested for evaluating DNA excision by PCR, expression of the integrated promoter sequence by luciferase assay, and expression of Cas9 by Western blot. Results from these experiments show the detection of a post-cleavage 205 bp DNA fragment in cells infected with HIV-1_JRFL_ and HIV-1_SF162_, indicating that production of Tat by HIV-1_JRFL_ and HIV-1_SF162_ transactivated the LTR_(−80/+66)_ promoter and production of Cas9 in these cells (Fig. 2b). It was also noted that the level of viral DNA cleavage by Cas9 under HIV-1_SF162_ infection is less than that seen in HIV-1_JRFL_. This difference may result from the variation in the level of infectivity of the cells by these two viral isolates, and/or the different levels of Tat production by these viruses and their potency in stimulating the minimal LTR promoter that initiates Cas9 expression in these cells. Results from luciferase assay revealed significant reduction of luciferase activity in the cells, again verifying the effectiveness of Cas9 activation by Tat, which is produced upon infection by HIV-1_JRFL_ or HIV-1_SF162_ in shutting down the integrated HIV-1 luciferase gene. Results from Western blot showed activation of the truncated LTR promoter, LTR_−80/+66_, upon infection of cells with HIV-1_JRFL_ and HIV-1_SF162_, resulting in the production of Cas9 protein in the cells (Fig. 2a).

**Figure 3:**
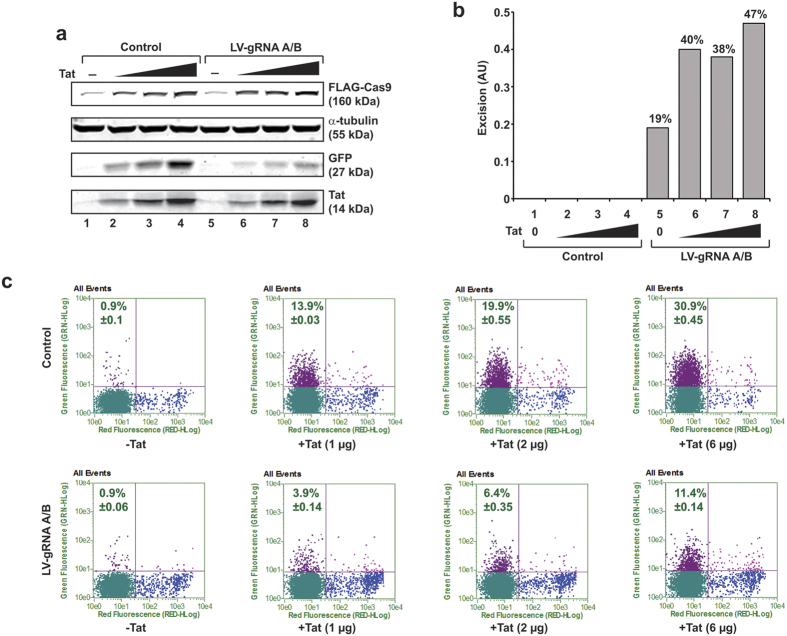
Tat stimulation of Cas9 cleaves integrated HIV-1 DNA in T-cells encompassing the HIV-1 reporter at a latent stage. 2D10 cells with integrated copies of LTR_−80/+66_-Cas9 gene were transfected with control (pKLV) or pKLV-gRNA A/B along with pCMV or pCMV-Tat plasmids. After 48 hours, the level of various proteins, as depicted, was determined by Western blot (**a**). The genomic DNA for assessing the state of the integrated HIV-1 DNA was determined by LTR specific PCR and the excision efficiency was determined as a percentage of ratios between truncated vs. full-length amplicon and presented in arbitrary units (AU) 0–0.5 (**b**). The level of integrated viral promoter reactivation after cleavage was assessed by flow cytometry and the representative scatter plots are shown (**c**). Red positive, propidium iodide labeled, dead cells were excluded from the analysis.

In a follow-up experiment, we tested the ability of Tat-mediated activation of the LTR-Cas9 along with gRNAs A/B in eliminating the HIV-1 genome in the human T-lymphocytic cells line, 2D10^21^. These cells harbor integrated copies of a single round HIV-1_NL4-3_ in a latent state, whose genome lacks a portion of the Gag and Pol genes and the Nef gene is replaced by a gene encoding the reporter green fluorescent protein (GFP). The enhanced level of Tat protein in these cells and the activation of Cas9 (shown in Fig. 3a) caused editing of the viral LTR upon activation of Cas9 in the cells transduced by LV-gRNAs A/B (Fig. 3b, also see Supplemental Fig. 2, lanes 1–8). Accordingly, a significant decrease in the number of GFP positive cells was detected in the presence of Tat (Fig. 3c), indicating that activation of Tat eliminates the capacity of the promoter in expressing viral DNA, which in turn, causes suppression of GFP in these cells. The DNA sequence corresponding to the position of the gRNAs, excision of the DNA fragment and PCR primers are shown in Supplemental Fig. 3. The basal level of Cas9 expression and viral DNA excision may attribute to the constitutive but lowest expression of Tat in the latent 2D10 cell line.

**Figure 4:**
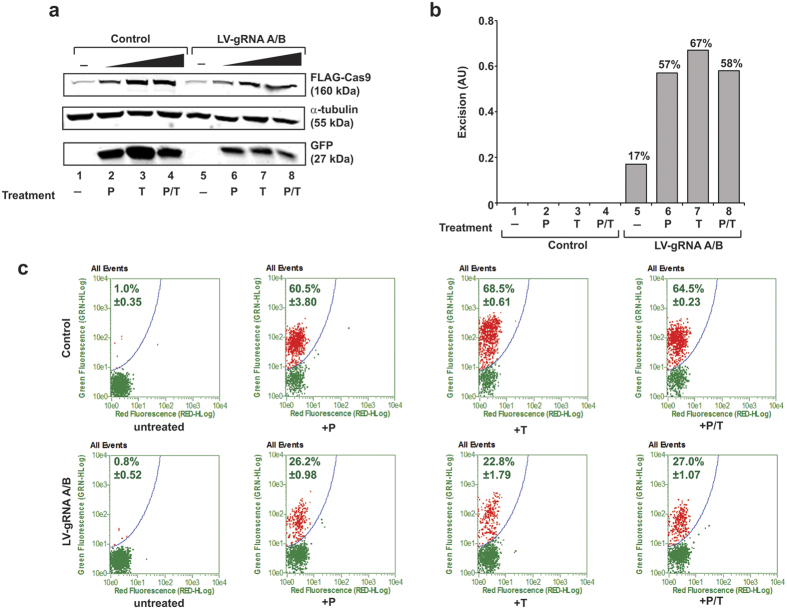
Treatment of cells with latency reversing drugs induces Cas9 expression and cleavage of integrated viral DNA in Jurkat 2D10 cells. 2D10 cells expressing LTR_−80/+66_-Cas9 were treated with control (empty) or lentivirus plasmid expressing gRNAs A/B and 24 hours later cells were treated with PMA (P), TSA (T) or both (P/T) for 16 hours, as indicated. Protein studies for the expression of Cas9-Flag, α-tubulin and GFP (indicative of the integrated HIV-1 genome) was determined by Western blot (**a**). Genomic DNA for the detection of the level of excision within the integrated LTR DNA by Cas9 and gRNA A/B was assessed by PCR and the excision efficiency was determined as described in Fig. 3 legend (**b**). GFP reporter assay, by flow cytometry, and representative scatter plot is shown (**c**).

In light of earlier observations indicating the ability of PMA and/or TSA in stimulating integrated copies of proviral DNA in 2D10 cells^21^, we sought to assess the impact of PMA and TSA on the activation of Cas9 in a latently infected T-cell model. As seen in Fig. 4a, treatment of 2D10 cells with PMA and TSA, singly or in combination, increased the level of Cas9 expression. In a parallel experiment, we performed PCR analysis for the detection of LTR DNA and showed a clear increase in the level of viral DNA excision (Fig. 4b), as evidenced by the appearance of the 227 bp DNA fragment (see Supplemental Fig. 2, lanes 9–14). Examination of viral activation by measuring the level of GFP in the cells using Western blot or the quantification of green fluorescent cells, indicative of viral activation, by flow cytometry (Fig. 4c) showed a drastic decrease in the level of viral gene expression. Thus, it is likely that production of Cas9 upon activation of the minimal viral promoter (−80/+66) by either Tat, which is expressed upon reactivation of the silent provirus DNA or by PMA and TSA, leads to editing of the integrated copies of viral DNA and exerts a negative effect on the expression of the latent viral genome in cells containing gRNAs A and B.

**Figure 5:**
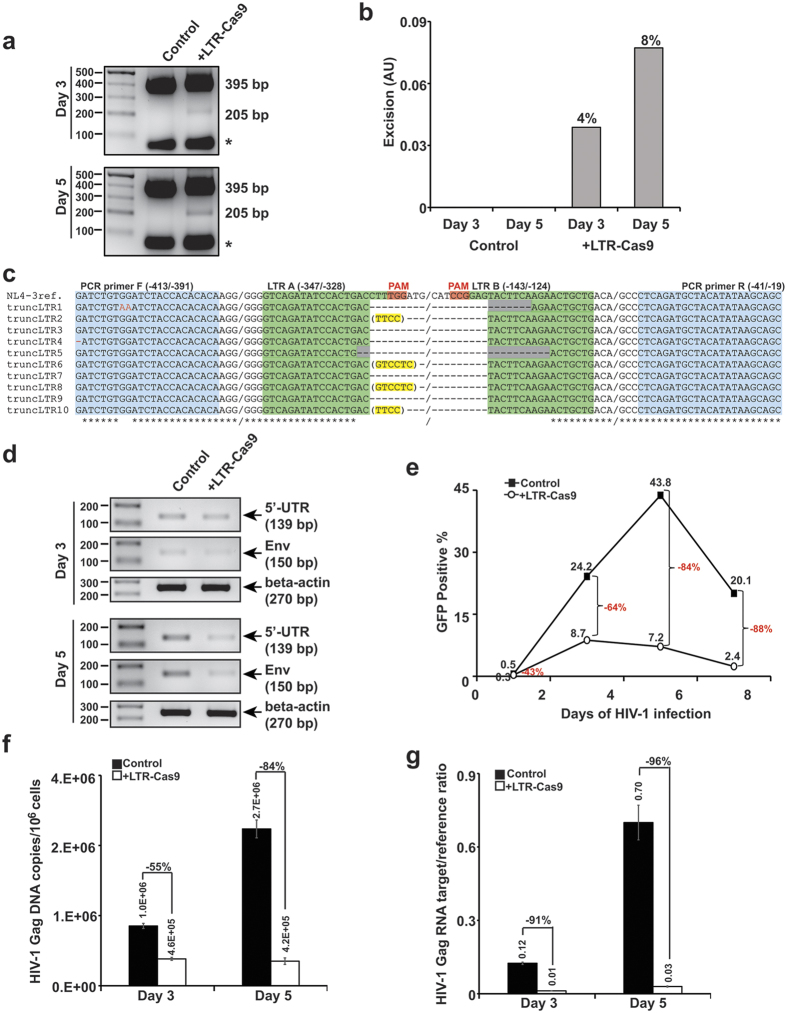
Expression of LTR-Cas9/gRNA protects cells from new HIV-1 infection. (**a,b**) Jurkat cells were co-transduced with LV-gRNA A/B and Lenti-LTR_(−80/+66)_-Cas9-Blast. The next day, cells were infected with HIV-1_NL4–3-GFP-P2A-Nef_ at MOI 0.01. At days 3 and 5 of infection cells were harvested and the level of excision was assessed by LTR specific PCR using genomic DNA as a template (Panel a) and quantified (Panel b) as in Fig. 3. (**c**) Sequencing analysis of the 205 bp DNA fragment after cloning in TA vector and selection of 10 clones designated at truncLTR 1–10, illustrating the positions of excision fragments compared to the control NL4–3. The positions of gRNAs corresponding to LTR A and LTR B as well as PAM sequences, and the primers used for amplification of the DNA are highlighted. (**d**) Agarose gels depicting results from PCR analysis for the DNA segment corresponding to 5′-UTR, Env, and control β-actin DNA in the experimental cells after 3 and 5 days of HIV-1 infection. (**e**) Results from flow cytometry quantifying the percentage of GFP-positive cells (indicative of viral expression) at three different times post infection. Quantitative detection of viral DNA **(f)** and viral RNA (**g**) corresponding to the Gag sequence by Taqman in which β-globin (for DNA) and β-actin (for RNA) were used as a reference. The asterisks (*) illustrate dimerized primers.

In the next series of experiments, we examined the level of HIV-1 replication in Jurkat T-cells containing LTR-Cas9. Cells were transduced with lentivirus vector (LV) expressing gRNAs A and B, and LTR_(−80/+66)_-Cas9. After 24 hours, the transduced cells were infected with HIV-1_NL4-3-EGFP-P2A-Nef_, and after 3 and 5 days, cells were harvested and viral DNAs were tested for the excision of a 190 bp DNA fragment spanning gRNAs A and B target sequences. As shown in Fig. 5 (Panels A and B), infection of cells with HIV-1 led to the appearance of a 205 bp amplicon in day 3 whose intensity was increased at day 5 of infection (Fig. 5a,b). This observation suggests that, similar to the results shown in Fig. 3, an increase in the level of Tat during the course of HIV-1 infection stimulated LTR-Cas9 expression, and hence, cleavage of LTR DNA. Direct sequencing of the 205 bp band seen in day 5 revealed cleavage sites within the LTR by Cas9/gRNA A and Cas9/gRNA B causing a range of InDel mutations that were detected in the junction of the 5′ and 3′ fusion sites (Fig. 5c). Examination of segments of the viral DNAs corresponding to the 5′-UTR (nt +97 to +235) and envelop (env) gene (nt +5828 to +5977), both of which are positioned between the 5′ and 3′ LTRs, showed a substantial decrease in the intensity of a 139 bp and 150 bp amplicons corresponding to the 5′-UTR and env gene, respectively at day 5 compared to day 3 (Fig. 5d). These observations suggest the excision of a larger DNA fragment of the HIV-1 genome spanning between the 5′ and 3′ LTRs upon cleavage by Cas9/gRNA A (at the 5′ LTR) and Cas9/gRNA B (at the 3′-LTR) may have also occurred upon treatment of the cells with Cas9 and gRNAs A and B, an event that has been reported previously^16^,^17^. Quantitative analysis of the results from flow cytometry illustrating expression of the reporter GFP, indicative of viral gene expression, showed substantial inhibition of GFP positive cells (64%) on day 3 and even more on day 5 (84%) and day 8 (88%). The presence of lentivirus harboring genes encoding gRNAs and the marker BFP and expression of GFP in the cells were monitored by fluorescent microscopy and the quality of cell cultures was tested by phase microscopy (Supplemental Figure S4A). Quantitative analysis showed that the total number of cells remained unchanged, indicating that similar to the previous observation^17^, no toxicity is associated with this excision strategy. In accord with results from PCR gel analysis (shown in Fig. 5d), results from qPCR and qRT-PCR showed a significant decrease in the level of viral DNA copy numbers corresponding to the Gag gene, i.e. 55% on day 3 and 84% on day 5 and Gag RNA level 91% on day 3 and 96% on day 5 post infection (Fig. 5f,g). We also performed a similar set of studies in human primary cultures of microglia and astrocytes. Results from these studies showed a significant suppression of viral gene expression and viral DNA presence in HIV-1 infected cells transduced with LVs expressing LTR-Cas9 and gRNAs (shown in Supplemental Fig. 5). Altogether, these observations provide evidence for the use of novel autoregulatory events by employing viral proteins, including Tat, to initiate the editing strategy using CRISPR/Cas9 by excising the viral genome and permanently suppressing viral replication.

## Discussion

**Figure 6:**
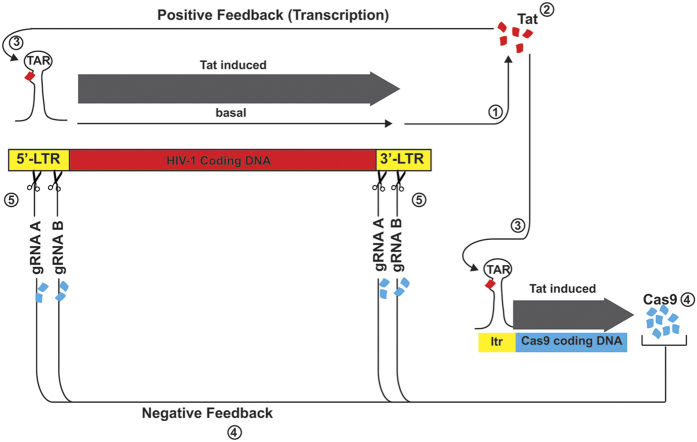
Schematic representation of negative feedback regulation of HIV-1 by CRISPR/Cas9. At the early stage of viral replication, basal transcription of the viral genome ➀ results in the production of Tat protein ➁. The association of Tat with the TAR stem loop structure within the leader of the viral transcript at the budge ➂ leads to the recruitment of several cellular regulatory proteins leading to the induction of viral transcription (solid thick arrow). At the early stage, Tat also stimulates the minimum viral promoter (depicted as ltr), which drives transcription of Cas9 gene ➃. The newly synthesized Cas9 upon association with the various HIV-1 specific gRNAs, in turn, cleaves the viral genome and permanently inactivates LTR activity by excising a large segment of viral DNA, hence shutting down HIV-1 gene expression and replication.

Since its discovery in 1985^22^,^23^, the Tat transactivator protein of HIV-1 has been shown to be a critical regulatory protein due to its role in expression of the viral genome at the transcriptional level and its pathogenic impact on uninfected cells. Mechanistically, Tat associates with the RNA sequence located downstream of the initiation site from transcription (nucleotides +1 to +59), the so-called transactivation responsive region or TAR. The association of Tat with TAR triggers a series of molecular and biochemical events leading to the formation of pre-initiation and initiation complexes of transcription in proximity to the transcription start site (nucleotide +1). This complex includes a series of cellular proteins that have the ability to phosphorylate or acetylate components of the complexes including pTEF and RNA polymerase II, thus facilitating transcriptional elongation of RNA (for review see)^24^,^25^. In addition, the interaction of Tat with various transcriptional factors including NF-κB^26^, p300/CBP and GCN5^27^,^28^,^29^ can affect transcription of other viral and cellular genes; all of which contribute to the disease spectrum seen in HIV-1 positive AIDS patients^30^. Tat also plays a major role in the productive replication of latent virus in reservoirs once transcription from the reactivated viral promoter leads to an initial round of viral transcription and Tat production. The unique importance of Tat in HIV-1 replication and the pathogenesis of AIDS, provided a strong rationale for serving as a potential target for drug discovery as well as vaccine development. In fact, several potent inhibitors, some with the ability to interfere with Tat-TAR interaction and others with the capacity to prevent Tat communication with its cellular partners, have shown various degrees of efficacy in affecting HIV-1 replication^31^. The strategy that we utilized in this study was to recruit Tat to stimulate Cas9 expression and promote excision of a segment of the viral genome and permanently ablate HIV-1 gene transcription and replication in cells with productive or latent HIV-1. Here we designed a suicide path for HIV-1 that is triggered by Tat and includes editing of the viral genome using CRISPR/Cas9 technology (illustrated in Fig. 6). According to this pathway, production of Tat in the cells, in addition to stimulating its own promoter with the full-length 5′-LTR sequence, potentiates expression of Cas9 through the same mechanism by a truncated minimal promoter sequence spanning the GC-rich, TATA box, and TAR (−80 to +66) regions. Production of Cas9 and its association with gRNAs designed to target the LTR DNA sequence outside of the (−80 to +66) induced InDel mutations within the full-length viral promoter and by excising a segment of the gene, can permanently eradicate HIV-1 in the T-cells. In addition to the expected 417 bp DNA fragment representing the full-length LTR sequence, results from short-range amplification of LTR DNA showed a second DNA fragment of 227 bp in size found only in cells expressing Tat. The 227 bp DNA fragment was generated by joining the residual 5′-LTR to the remaining 3′-LTR after cleavage by Cas9/gRNA A at either the 5′-LTR or the 3′-LTR. It is also likely that ligation of the remaining DNA fragment from the 5′-LTR with those from the 3′-LTR after cleavage by Cas9/gRNA created a new template for gene amplification and the appearance of a 227 bp amplicon. As noted in this study, we aimed to target the sequences positioned between nucleotides −347 to −328 and −143 to −124 by gRNAs A and B, respectively. In earlier studies we demonstrated the safety of HIV-1 DNA cleavage by targeting these two regions of HIV-1 by CRISPR technology, we reported no off-target effects on the cellular genes by a variety of methods including deep sequencing and bioinformatic studies^17^.

Our results demonstrate that the level of viral DNA excision at the early stage of infection is low and as infection continues, more viral DNA is being edited by Cas9 as evidenced by the results from conventional PCR, flow cytometry as well as Taqman quantitative PCR for detection of viral DNA copy numbers. The low level of viral DNA excision at the early stage of infection, i.e. day 3, may be attributed to the low levels of Tat expression and/or engagement of Cas9 with large numbers of non-integrating viral DNAs that are produced at the early phase of infection by reverse transcription. However, at the later stages, once copies of viral DNA are integrated in the host chromosome, the coordinate utilization of multiplex of gRNAs, in this case gRNA A and B, by Cas9 results in the excision of the viral genome and suppression of viral replication in the cells.

The CRISPR/Cas9 gene editing strategy has received attention in biomedical research in recent years due to its extraordinary ability to edit the genome with precision and high efficiency and its simplicity and flexibility of implementation. However, there are several areas that need close attention. For example, it is important to design the most specific and effective gRNAs to avoid off-target effects. The strategy that we have employed for maximizing specificity and avoiding off-target editing was verified by ultra deep sequencing of the whole genome and various other tests, as described^16^,^17^. Treatment of the cells by a single RNA may lead to the development of mutant HIV-1 as a result of unfaithful NHEJ repair at the site of cleavage, and potentially lead to the emergence of mutant virus that becomes resistant to the initial single gRNA^32^,^33^. Employment of multiplex of gRNAs, which, by introducing multiplex double-strand breaks across the HIV-1 genome, leads to the excision of a larger segment of viral DNA from the host genome, alleviating this concern and permanently eliminating any chance for the emergence of replication-competent virus^13^,^15^,^16^,^17^,^18^,^19^. The second issue relates to the controlled expression of Cas9 to avoid the unnecessary persistence of expression of the protein that may non-specifically cause injury to the host genome in the long term and/or induce an immune response. Our strategy for conditional expression of Cas9, by HIV-1 Tat, may provide a novel approach for stimulating the silent gene editing molecule to be expressed and to excise HIV-1 DNA at the early stage of virus reactivation. Indeed, several alternative strategies, including Rev/RRE, can independently or in combination with Tat, can be utilized for controlling CRISPR/Cas9 expression in HIV-1 infected cells.

## Materials and Methods

### Plasmid preparation

Full length and truncated LTR promoter sequences were obtained by PCR using pNL4-3 HIV vector (NIH AIDS Reagent Program #114) as a template and the primers are listed in Table SI.1. PCR products were gel purified and directly subcloned in TA vector (Invitrogen), then excised with Kpn1 or Xba1 and Nco1 restriction enzymes and ligated into Kpn1-Nco1 or Xba1-Nco1 digested pX260-U6-DR-BB-DR-Cbh-NLS-hSpCas9-NLS-H1-shorttracr-PGK-puro plasmid (Addgene #42229). As a result, the original Cbh promoter (Xba1-Kpn1-Cbh-Nco1) in pX260 plasmid was removed and replaced with one of the LTR promoters (Xba1- or Kpn1-LTR-Nco1). To create a lentiviral LTR-Cas9 construct, lentiCas9-Blast (Addgene #52962) was treated with Nhe1/Xba1, as a result, EFS promoter sequence was removed and replaced by Nhe1-LTR−80/+66-Xba1 digested PCR product creating Lenti-LTR−80/+66-Cas9-Blast plasmid (Table SI.2). pKLV-U6-LTR A/B-PGKpuro2ABFP lentiviral plasmid was described previously^17^. pcDNA3.1 control vector purchased from Invitrogen, and pCMV-Tat86 has been described previously^34^. pKLV-U6-LTR A/B-PGKpuro2ABFP were packaged into lentiviral particles by co-transfection of HEK293T cells with pMDLg/pRRE (Addgene 12251), pRSV-Rev (Addgene 12253) and pCMV-VSV-G (Addgene 8454). For packaging Cas9 into lentiviral particles following vectors were used: Lenti-LTR−80/+66-Cas9-Blast, psPAX2 (Addgene 12260) and pCMV-VSV-G (Addgene 8454).

### Cell culture and transient transfection

The TZM-bl reporter cell line was obtained from the National Institutes of Health (NIH) AIDS Reagent Program, Division of AIDS, National Institute of Allergy and Infectious Diseases, NIH. Jurkat and Clone E6 1 were purchased from ATCC (TIB-152™) Jurkat 2D10 reporter cell line is described previously^21^. TZM-bl cells were cultured in DMEM high glucose complemented with 10% FBS and gentamicin (10 ug/ml). Jurkat and Jurkat 2D10 cells were cultured in RPMI medium containing 10% FBS and gentamicin (10 ug/ml). Primary culture of human astrocytes and microglia were obtained from the Tissue Culture Core facility of the Comprehensive NeuroAIDS Center (CNAC) in the Department of Neuroscience at the Lewis Katz School of Medicine at Temple University in Philadelphia. In transfection experiments 2D10 cell encompassing LTR_(−80/+66)_-Cas9 were electroporated using Neon system (Invitrogen) with the following plasmids: control pKLV-gRNA-empty (6 ug) or pKLV-gRNA LTR A and B (3 ug of each) alone or together with 0 ug, 1 ug, 2 ug, 6 ug of pCMV-Tat86. Total amount of plasmid DNA used in transfections was kept equal (12 ug) with empty pCMV vector (pcDNA3.1). Electroporation conditions: 4 × 10^6^ cells/80 ul of buffer T/12 ug total plasmid DNA, 3 times 10 ms/1350 V impulse using 10 ul tips.

### Stable cell lines and subcloning

TZM-bl cells were plated in 6 well plates at 1 × 10^5^ cells/well and transfected using Lipofectamine 2000 reagent (Invitrogen) with 1 ug of pX260-LTR_(−80/+66)_-Cas9 plasmid. Next day cells were transferred into 100-mm dishes and cultured in the presence of puromycin (Sigma) at concentration 1 ug/ml. After two weeks surviving clones were isolated using cloning cylinders (Corning). Two million Jurkat 2D10 cells were electroporated with 10 ug pX260-LTR_(−80/+66)_-Cas9 plasmid (Neon System, Invitrogen, 3 times 10 ms/1350 V impulse). Forty-eight hours later medium was replaced with medium containing puromycin 0.5 ug/ml. After one week selection puromycin was removed and cells were allowed to grow for another week. Next, cells were diluted to a concentration of 10 cells/ml plated in 96 well plates, 50 ul/well and cultured for 2 weeks. Both TZM-bl and Jurkat 2D10 pX260-LTR_(−80/+66)_-Cas9 single cell clones were screened for Cas9-FLAG expression after transfections with control pCMV-empty (pcDNA3.1) or pCMV-Tat plasmids by Western blot. Single cell clones with undetectable/very low level of Cas9 under control conditions and very high levels upon Tat overexpression were expanded and used in further experiments.

### Lentivirus packaging

HEK 293T cells were co-transfected using CaPO_4_ precipitation method in the presence of chloroquine (50 uM) with packaging lentiviral vectors mixtures at 30 ug total DNA/2. 5 × 10^6^ cells/100 mm dish. Next day medium was replaced and 24 and 48 h later supernatants were collected, clarified at 3000 RPM for 10 minutes, 0.45 um filtered and concentrated by ultracentrifugation (2 h, 25000 RPMI, with 20% sucrose cushion). Lentiviral pellets were resuspended in HBSS by gentle agitation overnight, aliquoted and tittered in HEK 293T cells. Lenti-LTR_(−80/+66)_Cas9-Blast lentivirus was tittered by FLAG immunocytochemistry, pKLV-U6-LTR A/B-PGKpuro2ABFP lentiviruses by BFP fluorescent microscopy.

### Viral stock

For creation of HIV-1_NL4-3-EFGP-p2A-Nef_, we used fusion PCR^35^ to amplify the EGFP gene, a P2A self-cleaving peptide^36^, and N-terminal of HIV-1 Nef in frame with HIV-1 splicing acceptor originally for HIV-1 Nef expression. DNA was then cloned into the BamHI and XholI restriction sites of the HIV-1 proviral clone pNL4–3^37^ obtained from Dr. Malcolm Martin through the NIH AIDS Reagent Program, Division of AIDS, NIAID, NIH. The self-cleaving P2A peptide from porcine teschovirus-1 between the GFP and Nef allows the expression of HIV-1 Nef in full length^38^.

HIV-1_NL4-3-EGFP-P2A-Nef_ reporter virus was prepared by transfecting HEK 293T cells with pNL4-3-EGFP-P2A-Nef plasmid (University of Pittsburgh School of Medicine) processed like lentiviral stocks (see above) and tittered by GFP-FACS in Jurkat cell line.

HIV-1 JRFL and SF162 crude stocks used was prepared from supernatants of PBMCs infected with HIV-1 for 6 days, clarified at 3000 RPM for 10 minutes and 0.45 um filtered. Virus was tittered using Gag p24 ELISA.

### *In vitro* HIV-1 infection

Jurkat cells were infected by spinoculation for 2 h at 2700 RPM, 32 °C in 500 ul inoculum containing 8 ug/ml polybrene then resuspended and left for 4 h, then 500 ul of growth medium was added. The next day cells were washed 3 times with PBS and resuspended in growth medium. For infection of astrocytes and microglial cells, primary human fetal brain cells were transduced/infected by incubation with viral stocks diluted in Opti-MEM medium in the presence of polybrene (8 ug/ml) for 4 h, then 1 ml of growth medium was added for overnight. The next day cells were washed 3 times with PBS and fresh grow medium was added.

### HIV-1 DNA detection and quantification

Genomic DNA was isolated from cells using NucleoSpin Tissue kit (Macherey-Nagel) according to the protocol of the manufacturer. For HIV-1 and β-actin specific PCRs (see Table SI.3), 100 ng of extracted DNA was subjected to PCR using Fail Safe PCR kit and buffer D (Epicentre) under the following PCR conditions: 98 °C 5 minutes, 30 cycles (98 °C 30 s, 55 °C 30 s, 72 °C 30 s), 72 °C 7 minutes and resolved in 2% agarose gel. PCR products were subjected to agarose gel electrophoresis, gel purified, cloned into TA vector (Invitrogen) and send for Sanger sequencing (Genewiz). HIV-1 DNA was quantified using TaqMan qPCR specific for HIV-1 5′-UTR and Env genes and cellular beta-globin gene as a reference (see Table SI.4). Prior to qPCR, genomic DNA from infected cells was diluted to 10 ng/ul and then 5 ul (=50 ng) was taken per reaction/well. Reaction mixtures were prepared using Platinum Taq DNA Polymerase (Invitrogen) according simplified procedure from Liszewski^39^. Standard was prepared from serial dilutions of U1 cells genomic DNA since it contains two single copies of HIV-1 provirus per diploid genome equal to beta-globin gene copy number. qPCR conditions: 98 °C 5 minutes, 45 cycles (98 °C 15 s, 62 °C 30 s with acquisition, 72 °C 1 minute). Reactions were carried out and data analyzed in a LightCycler480 (Roche).

### Reverse transcription and PCR

Total RNA was extracted from cells using RNeasy kit (Qiagen) with on column DNAse I digestion. Next 1 ug of RNA was used for M-MLV reverse transcription reactions (Invitrogen). cDNA was diluted and quantified using TaqMan qPCR specific for HIV-1 Gag and Env genes and cellular beta-actin gene as a reference (Table SI.4) under the same protocol like genomic DNA but analyzed using relative quantification mode.

### Flow cytometry and cell viability assay

GFP expression in 2D10 cells was quantified in live cells using Guava EasyCyte Mini flow cytometer (Guava Technologies). Cell viability was assessed using propidium iodide (PI) staining. To 200 ul of live cells 10^5^ in suspension PI solution was added to final concentration 10 ug/ml. Samples were incubated for 5 minutes at room temperature in the dark. After incubation, samples were acquired using a Guava EasyCyte Mini flow cytometer. In HIV-1_NL4-3-GFP-P2A-Nef_ infected Jurkat cells, first cells were fixed for 10 minutes in 2% paraformaldehyde then washed 3 times in PBS. For analysis of GFP in the fixed cells we used Guava EasyCyte mini flow cytometry.

### Western-blot

Whole cell lysates were prepared by incubation of Jurkat cells in TNN buffer [50 mM Tris pH 7.4, 150 mM NaCl, 1% Nonidet P-40, 5 mM EDTA pH 8, 1x protease inhibitor cocktail for mammalian cells (Sigma)] for 30 minutes on ice then pre-cleared by spinning at top speed for 10 minutes at 4 °C. Fifty micrograms of lysates were denatured in 1x Laemli buffer and separated by SDS-polyacrylamide gel electrophoresis in Tris-glycine buffer followed by transfer onto nitrocellulose membrane (BioRad). The membrane was blocked in 5% milk/PBST for 1 h and then incubated with mouse anti-flag M2 monoclonal antibody (1:1000, Sigma) or mouse anti-α-tubulin monoclonal antibody (1:2000). After washing with PBST, the membranes were incubated with conjugated goat anti-mouse antibody (1:10,000) for 1 h at room temperature. The membrane was scanned and analyzed using an Odyssey Infrared Imaging System (LI-COR Biosciences).

## Additional Information

**How to cite this article**: Kaminski, R. *et al*. Negative Feedback Regulation of HIV-1 by Gene Editing Strategy. *Sci. Rep.* **6**, 31527; doi: 10.1038/srep31527 (2016).

## Supplementary Material

Supplementary Information

## References

[b1] Alimonti J. B., Ball T. B. & Fowke K. R. Mechanisms of CD4+ T lymphocyte cell death in human immunodeficiency virus infection and AIDS. J. Gen. Virol. 84, 1649–1661 (2003). 12810858 10.1099/vir.0.19110-0

[b2] Okoye A. A. & Picker L. J. CD4(+) T-cell depletion in HIV infection: mechanisms of immunological failure. Immunol Rev 254, 54–64 (2013). 23772614 10.1111/imr.12066 PMC3729334

[b3] WHO. 2015 “About HIV/AIDS”. CDC. December 6, 2015. http://www.cdc.gov/hiv/basics/whatishiv.html (Retrieved April 1, 2016). “HIV/AIDS Fact sheet N°360”. WHO. November 2015. http://www.who.int/mediacentre/factsheets/fs360/en/ (Retrieved April 1, 2016).

[b4] Garcia J. A. . Human immunodeficiency virus type 1 LTR TATA and TAR region sequences required for transcriptional regulation. Embo j. 8, 765–778 (1989). 2721501 10.1002/j.1460-2075.1989.tb03437.x PMC400873

[b5] Reddy E. P. & Dasgupta P. Regulation of HIV-1 gene expression by cellular transcription factors. Pathobiology 60, 219–224 (1992). 1388719 10.1159/000163726

[b6] Marcello A., Zoppé M. & Giacca M. Multiple modes of transcriptional regulation by the HIV-1 Tat transactivator. Iubmb Life 51, 175–181 (2001). 11547919 10.1080/152165401753544241

[b7] Roebuck K. A. & Saifuddin M. Regulation of HIV-1 transcription. Gene Expr 8, 67–84 (1999). 10551796 PMC6157391

[b8] Hatano H. . Evidence of persistent low-level viremia in long-term HAART-suppressed, HIV-infected individuals. Aids 24, 2535–2539 (2010). 20651585 10.1097/QAD.0b013e32833dba03 PMC2954261

[b9] Pasternak A. O. . Highly sensitive methods based on seminested real-time reverse transcription-PCR for quantitation of human immunodeficiency virus type 1 unspliced and multiply spliced RNA and proviral DNA. J. Clin. Microbiol. 46, 2206–2211 (2008). 18463204 10.1128/JCM.00055-08 PMC2446885

[b10] Archin N. M. . HIV-1 expression within resting CD4+ T cells after multiple doses of vorinostat. J. Infect. Dis. 210, 728–735 (2014). 24620025 10.1093/infdis/jiu155 PMC4148603

[b11] Manson McManamy M. E., Hakre S., Verdin E. M. & Margolis D. M. Therapy for latent HIV-1 infection: the role of histone deacetylase inhibitors. Antivir. Chem. Chemother. 23, 145–149 (2014). 24318952 10.3851/IMP2551 PMC3947511

[b12] Siliciano J. D. & Siliciano R. F. Recent developments in the search for a cure for HIV-1 infection: targeting the latent reservoir for HIV-1. J. Allergy Clin. Immunol. 134, 12–19 (2014). 25117799 10.1016/j.jaci.2014.05.026

[b13] Khalili K., Kaminski R., Gordon J., Cosentino L. & Hu W. Genome editing strategies: potential tools for eradicating HIV-1/AIDS. J. Neurovirol. 21, 310–321 (2015). 25716921 10.1007/s13365-014-0308-9 PMC4433555

[b14] White M. K., Hu W. & Khalili K. The CRISPR/Cas9 genome editing methodology as a weapon against human viruses. Discov. Med. 19, 255–262 (2015). 25977188 PMC4445958

[b15] Yin C. . Functional screening of guide RNAs targeting the regulatory and structural HIV-1 viral genome for a cure of AIDS. Aids 30, 1163–1170 (2016). 26990633 10.1097/QAD.0000000000001079 PMC4851589

[b16] Hu W. . RNA-directed gene editing specifically eradicates latent and prevents new HIV-1 infection. Proc. Natl. Acad. Sci. USA 111, 11461–11466 (2014). 25049410 10.1073/pnas.1405186111 PMC4128125

[b17] Kaminski R. . Elimination of HIV-1 Genomes from human T-lymphoid cells by CRISPR/Cas9 gene editing. Sci. Rep. 6, 22555 (2016). 26939770 10.1038/srep22555 PMC4778041

[b18] Ebina H., Misawa N., Kanemura Y. & Koyanagi Y. Harnessing the CRISPR/Cas9 system to disrupt latent HIV-1 provirus. Sci. Rep. 3, 2510 (2013). 23974631 10.1038/srep02510 PMC3752613

[b19] Liao H. K. . Use of the CRISPR/Cas9 system as an intracellular defense againse HIV-1 infectio nin human cells. Nature Comm . 6, 6413 (2015). 10.1038/ncomms7413 25752527

[b20] Karpinski J. . Directed evolution of a recombinase that excises the provirus of most HIV-1 primary isolates with high specificity. Nat. Biotechnol. 34, 401–409 (2016). 26900663 10.1038/nbt.3467

[b21] Pearson R. . Epigenetic silencing of human immunodeficiency virus (HIV) transcription by formation of restrictive chromatin structures at the viral long terminal repeat drives the progressive entry of HIV into latency. J. Virol. 82: 12291–12303 (2008). 18829756 10.1128/JVI.01383-08 PMC2593349

[b22] Arya S. K., Guo C., Josephs S. F. & Wong-Staal F. Trans-activator gene of human T-lymphotropic virus type III (HTLV-III). Science 229, 69–73 (1985). 2990040 10.1126/science.2990040

[b23] Sodroski J. . Trans-acting transcriptional regulation of human T-cell leukemia virus type III long terminal repeat. Science 227, 171–173 (1985). 2981427 10.1126/science.2981427

[b24] Mbonye U. & Karn J. Transcriptional control of HIV latency: cellular signaling pathways, epigenetics, happenstance and the hope for a cure. Virology 454–455, 328–339 (2014). 10.1016/j.virol.2014.02.008 PMC4010583 24565118

[b25] Taube. R. & Peterlin M. Lost in transcription: molecular mechanisms that control HIV latency. Viruses 5, 902–927 (2013). 23518577 10.3390/v5030902 PMC3705304

[b26] Taylor J. P. & Khalili K. Activation of HIV-1 transcription by Tat in cells derived from the CNS: evidence for the participation of NF-kappa B–a review. Adv. Neuroimmunol. 4, 291–303 (1994). 7874398 10.1016/s0960-5428(06)80270-6

[b27] Colm E. . The histone acetyltransferase, hGCN5, interacts with and acetylates the HIV transactivator, Tat. J. Biol. Chem. 276, 28179–28184 (2001). 11384967 10.1074/jbc.M101385200

[b28] Kiernan R. E. . HIV-1 tat transcriptional activity is regulated by acetylation. Embo j. 18, 6106–6118 (1999). 10545121 10.1093/emboj/18.21.6106 PMC1171675

[b29] Ott M. . Acetylation of the HIV-1 Tat protein by p300 is important for its transcriptional activity. Curr. Biol. 9, 1489–1492 (1999). 10607594 10.1016/s0960-9822(00)80120-7

[b30] Gibellini D., Vitone F., Schiavone P. & Re M. C. HIV-1 tat protein and cell proliferation and survival: a brief review. New Microbiol. 28, 95–109 (2005). 16035254

[b31] Tabarrini. O., Desantis J. & Massari S. Recent advances in the identification of Tat-mediated transactivation inhibitors: progressing toward a functional cure of HIV. Future Med Chem 8, 421–442 (2016). 26933891 10.4155/fmc.16.3

[b32] Wang G., Zhao N., Berkhout B. & Dat A. T. CRISPR-Cas9 can inhibit HIV-1 replication by NHEJ repair facilitates virus escape. Mol. Ther. 24, 522–526 (2016a). 26796669 10.1038/mt.2016.24 PMC4786927

[b33] Wang Z. . CRISPR/Cas9-derived mutations both inhibit HIV-1 replication and accelerate viral escape. Cell Rep. 15, 481–489 (2016b). 27068471 10.1016/j.celrep.2016.03.042

[b34] Gallia G. L. . Association of HIV-1 Tat with the cellular protein, Purα, is mediated by RNA. Proc. Natl. Acad. Sci. USA 96, 11572–11577 (1999). 10500218 10.1073/pnas.96.20.11572 PMC18075

[b35] Heckman K. L. & Pease L. R. Gene splicing and mutagenesis by PCR-driven overlap extension. Nat. Protoc. 2, 924–932 (2007). 17446874 10.1038/nprot.2007.132

[b36] Kim J. H. . High cleavage efficiency of a 2A peptide derived from porcine teschovirus-1 in human cell lines, zebrafish and mice. PLoS One 6, e18556 (2011). 21602908 10.1371/journal.pone.0018556 PMC3084703

[b37] Adachi A. . Production of acquired immunodeficiency syndrome-associated retrovirus in human and nonhuman cells transfected with an infectious molecular clone. J. Virol. 59, 284–291 (1986). 3016298 10.1128/jvi.59.2.284-291.1986 PMC253077

[b38] Edmonds T. G. . Replication competent molecular clones of HIV-1 expressing Renilla luciferase facilitate the analysis of antibody inhibition in PBMC. Virology 408, 1–13 (2010). 20863545 10.1016/j.virol.2010.08.028 PMC2993081

[b39] Liszewski M. K., Yu J. J. & O’Doherty U. Detecting HIV-1 integration by repetitive-sampling Alu-gag PCR. Methods 47, 254–260 (2009). 19195495 10.1016/j.ymeth.2009.01.002 PMC2862469

