# Supplementary material for: Negative Feedback Regulation of HIV-1 by Gene Editing Strategy
Source: Sci Rep. 2016 Aug 16;6:31527. doi: 10.1038/srep31527 (PMC4985742; doi:10.1038/srep31527)
Supplement: Supplementary Information [file srep31527-s1.pdf]

7/13/16

SUPPLEMENTARY FIGURES

Negative Feedback Regulation of HIV-1 by Gene Editing Strategy

Rafal Kaminski[1†], , Yilan Chen[1], Julian Salkind[1], Ramona Bella[1] Won-bin Young[2], Pasquale Ferrante[1,3], Jonathan Karn[4], Thomas Malcolm[5], Wenhui Hu[1†], Kamel Khalili[1†]

1. Department of Neuroscience
Center for Neurovirology
Lewis Katz School of Medicine at Temple University
3500 N. Broad Street, 7th Floor
Philadelphia, PA 19140

2. Department of Radiology
University of Pittsburgh School of Medicine
Pittsburgh, PA 15219

3. Microbiology and Clinical Microbiology
Department of Biomedical, Surgical and Dental Sciences
University of Milan
Milan, Italy

4. Department of Molecular Biology and Microbiology
Case Western Reserve University
Cleveland, OH 44106, USA

5. Excision Biotherapeutics, Inc.
3624 Market Street, #514
Philadelphia, PA 19104

†Corresponding author
Phone: 215.707.5192; Fax: 215.707.4888
Email: kamel.khalili@temple.edu (senior corresponding author)
wenhui.hu@temple.edu
rafalkim@temple.edu

Table SI

primer	sequence
**1. Cloning pX260-LTR-Cas9 constructs**	
**Kpn1**-LTR(-454)-F	5'-**GGTACC**TGGAAGGGCTAATTTGG-3'
**Kpn1**-LTR(-120)-F	5'**-GGTACC**TCGAGCTTTCTACAAGG-3'
**Xba1**-LTR(-80)-F	5'**-TCTAGA**GGAGGTGTGGCCTGGGC-3'
LTR(+66)-**Nco1**-R	5'**-CCATGG**TAAGCAGTGGGTTCC-3'
**2. Cloning lentiLTR(-80/+66)-Cas9-Blast construct**	
**Nhe1**-LTR(-80)-F	5'**-GCTAGC**GGAGGTGTGGCCTGGGC-3'
LTR(+66)-**Xba1**-R	5'**-TCTAGA**TAAGCAGTGGGTTCC-3'
**3. PCRs**	
LTR -417/F	5'-GATCTGTGGATCTACCACACACA-3'
LTR -19/R	5'-GCTGCTTATATGTAGCATCTGAG-3'
LTR -374/F	5'-TTAGCAGAACTACACACCAGGGCC-3'
LTR +43/R	5'-CCGAGAGCTCCCAGGCTCAGATCT-3'
HIV-1 5'UTR +97/F	5'-AAGTAGTGTGTGCCCGTCTG-3'
HIV-1 5'UTR +235/R	5'-TCGAGAGATCTCCTCTGGCT-3'
HIV-1 Env +5828/F	5'- TCCTTGGGATGTTGATGATCT-3'
HIV-1 Env +5977/R	5'- TGGCCCAAACATTATGTACC-3'
b- actin/F	5'-CTACAATGAGCTGCGTGTGGC-3'
b-actin/R	5'-CAGGTCCAGACGCAGGATGGC-3'
**4. Taqman qPCRs**	
HIV-1 5'UTR F	5'-AAGTAGTGTGTGCCCGTCTG-3'
HIV-1 5'UTR R	5'-TCGAGAGATCTCCTCTGGCT-3'
HIV-1 5'UTR probe	5'-FAM-CTGTTCGGGCGCCACTGCTA-ZEN-IowaBlackFQ-3'
HIV-1 env F	5'- TCCTTGGGATGTTGATGATCT-3'
HIV-1 env F	5'- TGGCCCAAACATTATGTACC-3'
HIV-1 env probe	5'-FAM-TGGTGGTTGCTTCTTTCCACACA-ZEN-IowaBlackFQ-3'
Hs b-globin F	5'-CCCTTGGACCCAGAGGTTCT-3'
Hs b-globin R	5'-CGAGCACTTTCTTGCCATGA-3'
Hs b-globin probe:	5'-FAM-GCGAGCATCTGTCCACTCCTGATGCTGTTATGGGCGCTCGC-ZEN-IowaBlackFQ-3'
Hs b-actin F	5'-TGGACTTCGAGCAAGAGATG-3'
Hs b-actin R	5'-GAAGGAAGGCTGGAAGAGTG-3'
Hs b-actin probe:	5'-FAM-CGGCTGCTTCCAGCTCCTCC-ZEN-IowaBlackFQ-3'

SUPPLEMENTAL FIGURE LEGENDS

Figure S1. Position and nucleotide sequences of gRNA A/B targets within the LTR (highlighted in green, PAM in red) and LTR specific primers used in PCR on TZM-bl and in vitro infected Jurkat cells genomic DNA (highlighted in blue) in the reference HIV-1 NL4-3 genome. Sequences and sizes of LTR specific PCR products (full-length and truncated) and predicted edited fragment.

Figure S2. A representative agarose gel analyzing LTR specific PCR reactions used for quantification of Cas9/gRNA mediated LTR excision efficiency in experiments using the Jurkat 2D10 reporter cell line from Figures 3 and 4.

Figure S3. Position and nucleotide composition of LTR gRNA A/B targets (highlighted in green, PAM in red) and LTR specific primers used to analyze excision by PCR in Jurkat 2D10 cells (highlighted in blue) in the reference HIV-1 NL4-3 genome. Nucleotide sequences and sizes of amplicons (full-length and truncated LTR DNA) and predicted excised DNA fragment are shown.

Figure S4. **(a)** A representative fluorescence microscopy images of tranduced/infected Jurkat cells at day 5 of infection. Expression of BFP is indicative of the presence a vector expressing gRNAs. HIV-1 infection was monitored by the leve of GFP. **(b)** Quantitative comparison of cell numbers at various time points between the control and experimental samples treated with LTR-Cas9.

Figure S5. Primary human fetal astrocytes and microglia were transduced with lentiviral cocktails containing: lenti-LTR-80/+66-Cas9 (MOI 10), lenti-KLV-BFP-LTR A, B (MOI 3.3 of each). At day 3 post-transduction cells were infected with HIV-1$_{NL4\text{-}3\text{-}GFP\text{-}P2A\text{-}Nef/VSV\text{-}G}$ at MOI 1. One week after HIV-1 infection cells were harvested and viral expression levels were quantified by GFP

expression in flow cytometry **(a)** viral DNA levels **(b)** and viral RNA **(c)** by Taqman qPCR and qRT-PCRs using primer set and probe specific for Gag gene.

Figure S1

TZMbl

F [-413/-391](T361)
TGGAAGGGCTAATTTGGTCCCAAAAAAGACAAGAGATCCTTGATCTGTGGATCTACCACACACAAGGCTA
LTR A [-347/-328]PAM
CTTCCCTGATTGGCAGAACTACACACCAGGGCCAGGGATCAGATATCCACTGACCTTTGGATGGTGCTTC
AAGTTAGTACCAGTTGAACCAGAGCAAGTAGAAGAGGCCAATGAAGGAGAGAACAACAGCTTGTTACACC
CTATGAGCCAGCATGGGATGGAGGACCCGGAGGGAGAAGTATTAGTGTGGAAGTTTGACAGCCTCCTAGC
PAM LTR B [-143/-124]
ATTTCGTCACATGGCCCGAGAGCTGCATCCGGAGTACTACAAAGACTGCTGACATCGAGCTTTCTACAAG
R [-41/-19](T363)
GGACTTTCCGCTGGGGACTTTCCAGGGAGGTGTGGCCTGGGCGGGACTGGGGAGTGGCGAGCCCTCAGAT
┌**Transcription start[+1]**
GCTACATATAAGCAGCTGCTTTTTGCCTGTACTG**G**GTCTCTCTGGTTAGACCAGATCTGAGCCTGGGAGC
TCTCTGGCTAACTAGGGAACCCACTGCTTAAGCCTCAATAAAGCTTGCCTTGAGTGCTCAAAGTAGTGTG

PCR product full length LTR -413/-19: 395bp

GATCTGTGGATCTACCACACACAAGGCTACTTCCCTGATTGGCAGAACTACACACCAGGGCCAGGGATCA
GATATCCACTGACCTTTGGATGGTGCTTCAAGTTAGTACCAGTTGAACCAGAGCAAGTAGAAGAGGCCAA
TGAAGGAGAGAACAACAGCTTGTTACACCCTATGAGCCAGCATGGGATGGAGGACCCGGAGGGAGAAGTA
TTAGTGTGGAAGTTTGACAGCCTCCTAGCATTTCGTCACATGGCCCGAGAGCTGCATCCGGAGTACTACA
AAGACTGCTGACATCGAGCTTTCTACAAGGGACTTTCCGCTGGGGACTTTCCAGGGAGGTGTGGCCTGGG
CGGGACTGGGGAGTGGCGAGCCCTCAGATGCTACATATAAGCAGC

+SpCas9/gRNA LTR A+LTR B:

GATCTGTGGATCTACCACACACAAGGCTACTTCCCTGATTGGCAGAACTACACACCAGGGCCAGGGATCA
GATATCCACTGAC

Edited fragment: 190bp

↓CTTTGGATGGTGCTTCAAGTTAGTACCAGTTGAACCAGAGCAAGTAGAAGAGGCCAATGAAGGAGAGAA
CAACAGCTTGTTACACCCTATGAGCCAGCATGGGATGGAGGACCCGGAGGGAGAAGTATTAGTGTGGAAG
TTTGACAGCCTCCTAGCATTTCGTCACATGGCCCGAGAGCTGCATCCGGAG↓

TACTACAAAGACTGCTGACATCGAGCTTTCTACAAGGGACTTTCCGCTGGGGACTTTCCAGGGAGGTGTG
GCCTGGGCGGGACTGGGGAGTGGCGAGCCCTCAGATGCTACATATAAGCAGC

PCR product truncated LTR -413/-19: 205bp

GATCTGTGGATCTACCACACACAAGGCTACTTCCCTGATTGGCAGAACTACACACCAGGGCCAGGGATCA
GATATCCACTGACTACTACAAAGACTGCTGACATCGAGCTTTCTACAAGGGACTTTCCGCTGGGGACTTT
CCAGGGAGGTGTGGCCTGGGCGGGACTGGGGAGTGGCGAGCCCTCAGATGCTACATATAAGCAGC

Figure S2

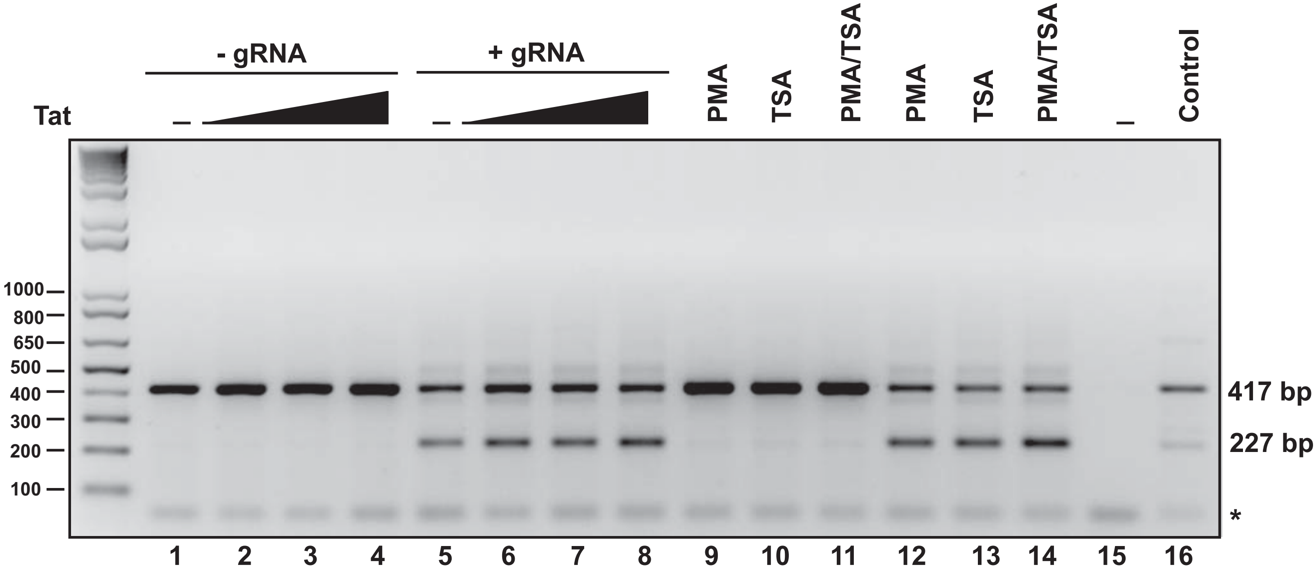

- gRNA
+ gRNA
Tat
PMA
TSA
PMA/TSA
PMA
TSA
PMA/TSA
Control
1000
800
650
500
400
300
200
100
417 bp
227 bp
*
1
2
3
4
5
6
7
8
9
10
11
12
13
14
15
16

Figure S3

Jurkat 2D10

Sequence:

TGGAAGGGCTAATTTGGTCCCAAAAAAGACAAGAGATCCTTGATCTGTGGATCTACCACACACAAGGCTA
F [-375/-352] **LTR A [-347/-328] PAM**
CTTCCCTGATTGGCAGAACTACACACCAGGGCCAGGGATCAGATATCCACTGACCTTTGGATGGTGCTTC
AAGTTAGTACCAGTTGAACCAGAGCAAGTAGAAGAGGCCAATGAAGGAGAGAACAACAGCTTGTTACACC
CTATGAGCCAGCATGGGATGGAGGACCCGGAGGGAGAAGTATTAGTGTGGAAGTTTGACAGCCTCCTAGC
PAM LTR B [-143/-124]
ATTTCGTCACATGGCCCGAGAGCTGCATCCGGAGTACTACAAAGACTGCTGACATCGAGCTTTCTACAAG
GGACTTTCCGCTGGGGACTTTCCAGGGAGGTGTGGCCTGGGCGGGACTGGGGAGTGGCGAGCCCTCAGAT
┌**Transcription start[+1]** R [+19/+43]
GCTACATATAAGCAGCTGCTTTTTGCCTGTACTG**G**GTCTCTCTGGTTAGACCAGATCTGAGCCTGGGAGC
TCTCTGGCTAACTAGGGAACCCACTGCTTAAGCCTCAATAAAGCTTGCCTTGAGTGCTCAAAGTAGTGTG

PCR product full length LTR -375/+43: 417bp

TTGGCAGAACTACACACCAGGGCCAGGGATCAGATATCCACTGACCTTTGGATGGTGCTTCAAGTTAGTA
CCAGTTGAACCAGAGCAAGTAGAAGAGGCCAATGAAGGAGAGAACAACAGCTTGTTACACCCTATGAGCC
AGCATGGGATGGAGGACCCGGAGGGAGAAGTATTAGTGTGGAAGTTTGACAGCCTCCTAGCATTTCGTCA
CATGGCCCGAGAGCTGCATCCGGAGTACTACAAAGACTGCTGACATCGAGCTTTCTACAAGGGACTTTCC
GCTGGGGACTTTCCAGGGAGGTGTGGCCTGGGCGGGACTGGGGAGTGGCGAGCCCTCAGATGCTACATAT
AAGCAGCTGCTTTTTGCCTGTACTG**G**GTCTCTCTGGTTAGACCAGATCTGAGCCTGGGAGCTCTCTGG

+SpCas9/gRNA LTR A+LTR B:

TTGGCAGAACTACACACCAGGGCCAGGGATCAGATATCCACTGAC

Edited fragment: 190bp

↓CTTTGGATGGTGCTTCAAGTTAGTACCAGTTGAACCAGAGCAAGTAGAAGAGGCCAATGAAGGAGAGAA
CAACAGCTTGTTACACCCTATGAGCCAGCATGGGATGGAGGACCCGGAGGGAGAAGTATTAGTGTGGAAG
TTTGACAGCCTCCTAGCATTTCGTCACATGGCCCGAGAGCTGCATCCGGAG↓

PCR product truncated LTR -375/-43: 227bp

TTGGCAGAACTACACACCAGGGCCAGGGATCAGATATCCACTGACTACTACAAAGACTGCTGACATCGAG
CTTTCTACAAGGGACTTTCCGCTGGGGACTTTCCAGGGAGGTGTGGCCTGGGCGGGACTGGGGAGTGGCG
AGCCCTCAGATGCTACATATAAGCAGCTGCTTTTTGCCTGTACTG**G**GTCTCTCTGGTTAGACCAGATCTG
AGCCTGGGAGCTCTCTGG

Figure S4

a

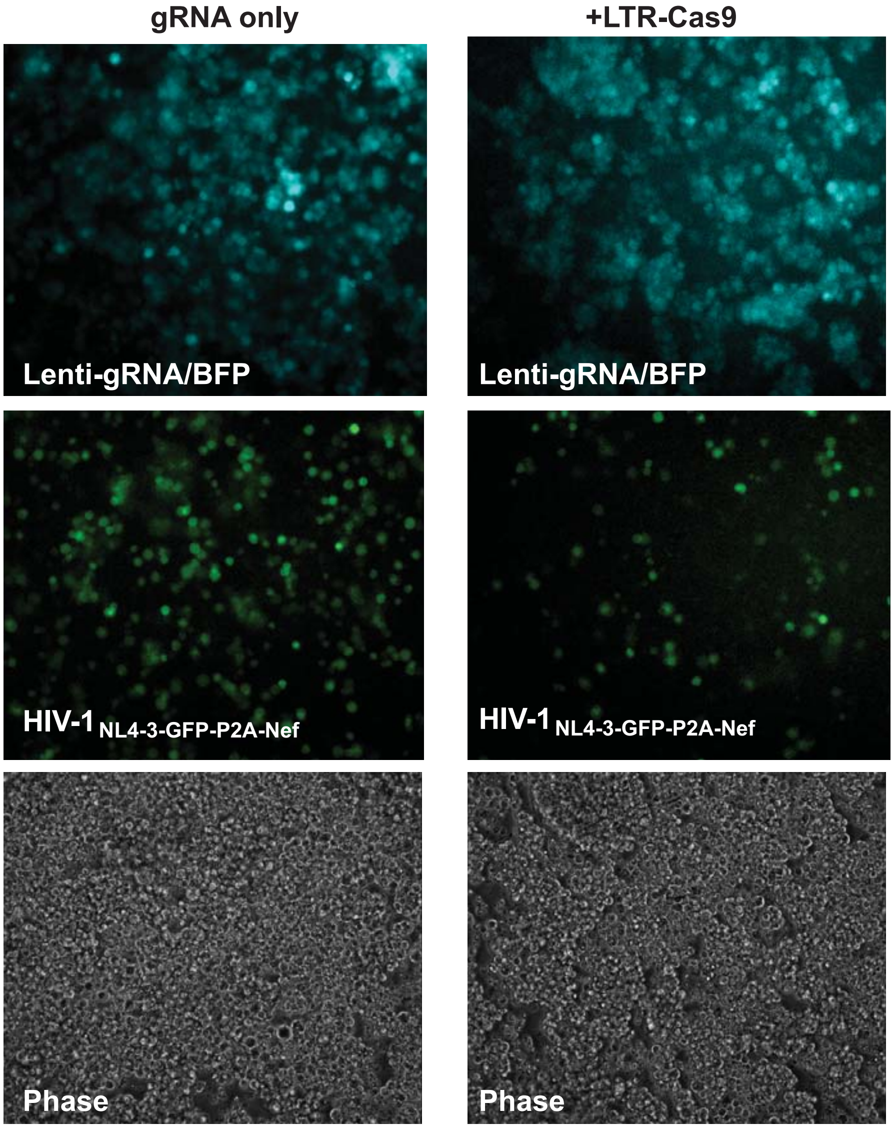
gRNA only
+LTR-Cas9
Lenti-gRNA/BFP
Lenti-gRNA/BFP
HIV-1 NL4-3-GFP-P2A-Nef
HIV-1 NL4-3-GFP-P2A-Nef
Phase
Phase

b

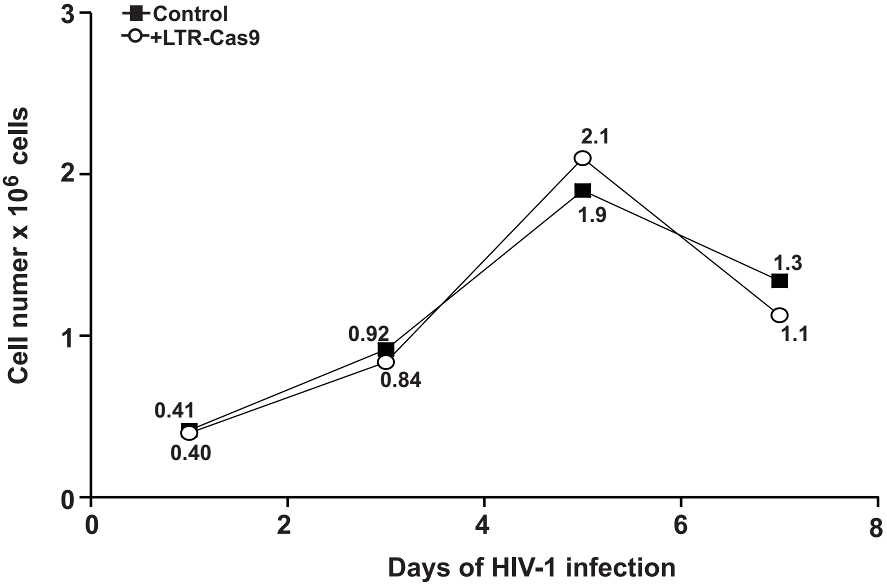
Control
+LTR-Cas9
Cell numer x 10^6 cells
Days of HIV-1 infection
0
1
2
3
0
2
4
6
8
0.41
0.40
0.92
0.84
2.1
1.9
1.3
1.1

Figure S5

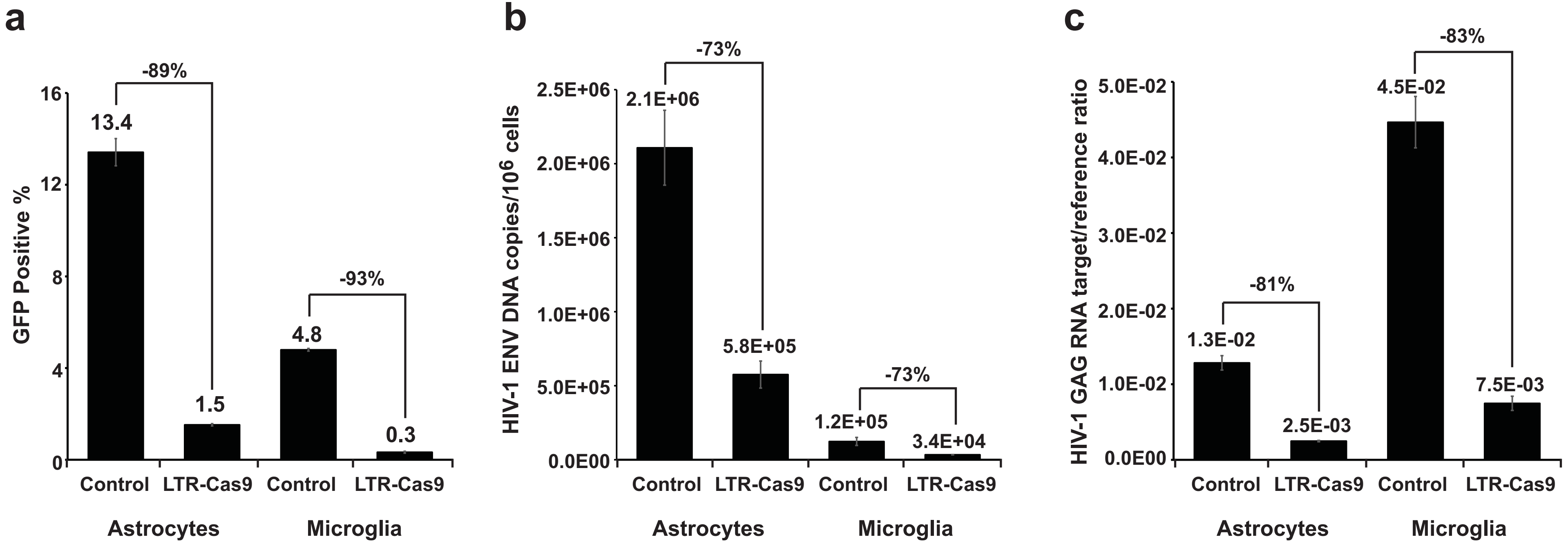
a
GFP Positive %
16
12
8
4
0
-89%
13.4
1.5
-93%
4.8
0.3
Control
LTR-Cas9
Control
LTR-Cas9
Astrocytes
Microglia
b
HIV-1 ENV DNA copies/10^6 cells
2.5E+06
2.0E+06
1.5E+06
1.0E+06
5.0E+05
0.0E00
-73%
2.1E+06
5.8E+05
-73%
1.2E+05
3.4E+04
Control
LTR-Cas9
Control
LTR-Cas9
Astrocytes
Microglia
c
HIV-1 GAG RNA target/reference ratio
5.0E-02
4.0E-02
3.0E-02
2.0E-02
1.0E-02
0.0E00
-83%
4.5E-02
7.5E-03
-81%
1.3E-02
2.5E-03
Control
LTR-Cas9
Control
LTR-Cas9
Astrocytes
Microglia
